# Intra-amniotic inflammation and child neurodevelopment: a systematic review protocol

**DOI:** 10.1186/s13643-018-0683-z

**Published:** 2018-01-22

**Authors:** Laurence Soucy-Giguère, Cédric Gasse, Yves Giguère, Suzanne Demers, Emmanuel Bujold, Amélie Boutin

**Affiliations:** 1 Reproduction, Mother and Child Health Unit, CHU de Québec-Université Laval Research Center, Université Laval, 2705, Boul. Laurier, TR-66, Québec, QC G1V 4G2 Canada; 2 Department of Social and Preventive Medicine, Faculty of Medicine, Université Laval, 1050, Avenue de la Médecine, Québec, QC G1V 0A6 Canada; 3 Department of Molecular Biology, Medical Biochemistry and Pathology, Faculty of Medicine, Université Laval, 1050, Avenue de la Médecine, Québec, QC G1V 0A6 Canada; 4 Department of Gynecology, Obstetrics and Reproduction, Faculty of Medicine, Université Laval, 1050, Avenue de la Médecine, Québec, QC G1V 0A6 Canada

**Keywords:** Intra-amniotic inflammation, Child neurodevelopment, Infants’ development

## Abstract

**Background:**

Intra-amniotic inflammation is associated with adverse pregnancy and neonatal outcomes. However, the impact on child neurodevelopment remains unclear. We aim to assess the effect of intra-amniotic inflammation on neurodevelopmental outcomes in children.

**Methods:**

The databases MEDLINE, Embase, CINAHL, and Cochrane will be searched from their inception until November 2017. Randomized trials and cohort studies in which inflammatory markers were measured in amniotic fluid collected by amniocentesis and in which infant’s neurodevelopment was assessed will be eligible. Two reviewers will independently select eligible studies, assess their risk of bias, and extract data. Results will be compared and a third party will be consulted in case of disagreement. Our primary outcome of interest is child neurodevelopment, assessed with either a validated tool or by revision of medical records for specific diagnosis. Secondary outcomes will include abnormal brain imaging. Relative risks will be pooled and sensitivity analyses will be performed for the indication of amniocentesis, gestational age at amniocentesis, gestational age at delivery, and fetal sex. Risk of bias will be assessed using the Cochrane Collaboration’s tool for assessing the risk of bias in randomized trials or an adapted version of the ROBINS-1 for the risk of bias in non-randomized studies.

**Discussion:**

This systematic review will report the current evidence regarding the association between amniotic inflammation and child neurodevelopment, and the modifiers of this association. The review will generate new hypotheses on pathological pathways and will guide future research.

**Systematic review registration:**

PROSPERO 2017 65065

**Electronic supplementary material:**

The online version of this article (10.1186/s13643-018-0683-z) contains supplementary material, which is available to authorized users.

## Background

Intra-amniotic infection and inflammation are frequently observed in women with preterm labor (PTL) or preterm premature rupture of membranes (PPROM), and both represent risk factors for adverse perinatal outcomes, such as an imminent delivery, early-onset sepsis, bronchopulmonary dysplasia, and intraventricular hemorrhage in the neonatal period [1–3]. It has been hypothesized that perinatal or neonatal inflammation may lead to brain damage and subsequent adverse neurodevelopmental outcomes [4–6]. Amniotic infection and chorioamnionitis are also associated with a greater risk of long-term neurodevelopment impairments, including encephalopathy, delayed motor development, and cerebral palsy in term and preterm infants [7–12]. Children born from mothers with a clinical or histologic diagnosis of chorioamnionitis are at increased risk of cerebral palsy [13, 14].

Yoon et al. observed in women with PTL that elevated concentrations of amniotic interleukin-6 or interleukin-1 beta, both indicators of intra-amniotic inflammation, were associated with an increased risk of white matter lesion in children. White matter lesions in the neonatal period, also known as periventricular leukomalacia, are important predictors of subsequent cerebral palsy [15]. Hence, these findings support the hypothesis of the contribution of intra-amniotic inflammation to the development of cerebral lesions [5]. Intra-amniotic inflammation, diagnosed either during a midtrimester genetic amniocentesis or at the time of PTL, has been associated with both adverse pregnancy and neonatal outcomes, including preterm delivery, and pulmonary, neurologic, gastro-intestinal, and infectious complications in preterm neonates [16–25]. Since cohort studies evaluating the association between intra-amniotic inflammation and subsequent child neurodevelopment are scarce, little is known about the long-term effect of intra-amniotic inflammation. Furthermore, it remains unclear when such infection or inflammation is initiated during pregnancy and whether the gestational age at diagnosis influences child neurodevelopment.

Of note, the interest for such systematic review has recently been enhanced by preclinical studies suggesting that antenatal suppression of interleukin-1 protects against inflammation-induced fetal injury, such as morphological alterations in the lung, intestine, and brain, and improved neonatal and developmental outcomes in mice [26]. As well, in pregnant women at high risk of preterm delivery, perinatal outcomes were improved with the use of antibiotics that cover better anaerobes and genital mycoplasmas, the most common pathogens involved in infection-related preterm birth [27].

Hence, there is a need to synthesize knowledge from studies on this topic to better understand the clinical significance of intra-amniotic inflammation on neurologic outcomes of children, including the role of gestational age and maternal condition at the time of diagnosis. We thus aim to assess the effect of amniotic inflammation on neurodevelopmental outcomes in children born either at term or preterm. We also aim to determine whether gestational age or pregnancy condition (ex: asymptomatic, with PTL, with PPROM) influences neurodevelopment.

## Methods

### Study design

We will conduct a systematic review of randomized trials and cohort studies that evaluated the association between amniotic inflammation and neurodevelopmental outcomes in children. The protocol of this systematic review follows the Preferred Reporting Items for Systematic Review and Meta-Analysis Protocols (PRISMA-P) guidelines [28] (Additional file 1) and is registered in PROSPERO (65065). The Cochrane Collaboration’s methodological recommendations and the Preferred Reporting Items for Systematic Reviews and Meta-Analyses (PRISMA) guidelines will be followed [29].

### Eligibility criteria

Published studies with the following criteria will be included in the review: (1) amniotic fluid collected by amniocentesis at any time during pregnancy; (2) measurement of any amniotic inflammatory marker; and (3) evaluation of child neurodevelopment assessed at any time-point, either with the use of a validated test or by revision of medical charts for specific diagnosis. No language restriction will be applied. A translation of articles in languages other than English, French, or Spanish will be obtained.

### Information sources

MEDLINE (PubMed), Embase, CINAHL, and the Cochrane Library will be searched from their inception until November 2017. Additionally, we will search conference proceedings published in the *Journal of Obstetrics and Gynaecology* of Canada, the *American Journal of Obstetrics and Gynecology*, *Obstetrics & Gynecology*, the *International Journal of Gynecology & Obstetrics*, the *American Journal of Perinatology*, the *Journal of Perinatal Medicine*, the *Journal of Maternal-Fetal and Neonatal Medicine*, the *British Journal of Obstetrics and Gynaecology*, *Pediatrics*, *and Paediatrics & Child Health*, over the last 5 years. In addition, references of selected publications will be screened to include potential articles not captured by electronic searches, and similarly, we will search articles citing the included studies for potential additional studies.

### Search strategy

To build the search strategy, MEDLINE and Embase were used to identify an extensive list of keywords, MeSH terms, and Emtrees that were related to the terms “amniotic fluid,” “inflammation,” and “cytokine.” The search strategy was revised and approved by all authors. The search strategy for MEDLINE (PubMed) is available in Table 1. A human studies filter will be applied. References will be managed with the software Endnote X8. Duplicates will be removed. References will then be exported to a Microsoft Excel (version 14.1.0, Redmond, WA: Microsoft, 2011) spreadsheet in order to complete the selection process.

**Table 1:** Search strategy for MEDLINE (PubMed)

Step	Search
1	cytokine[mesh] OR cytokine OR cytokines OR interleukin OR interleukins OR chemokines OR chemokine OR interferon OR interferons OR “tumor necrosis factor” OR macrophage OR “growth factor” OR “growth factors” OR lymphokine OR lymphokines OR metallopeptidase OR metallopeptidases OR metalloprotease OR metalloproteases OR metalloproteinase OR metalloproteinases OR “C reactive protein” OR “C-reactive protein” OR Inflammation OR inflammatory OR proinflammatory
2	“Amniocentesis”[Mesh] OR “Amniotic Fluid”[Mesh] OR amniot*[tiab] OR amniocent*[tiab] OR intra-amniot*[tiab] OR intraamniot*[tiab]
3	#1 AND #2
4	(animals[mesh] NOT humans[mesh])
5	#3 NOT #4

### Selection process

Titles and abstracts will be screened by two independent reviewers (L.S.G. and C.G.) to evaluate potential eligibility in the study. The same reviewers will check independently the full articles of the remaining titles and abstracts to verify whether they correspond to the inclusion criteria. In case of disagreement, a third party with expertise in maternal-fetal medicine and perinatal epidemiology will be consulted (E.B.).

### Data collection process

For all selected articles, two reviewers (L.S.G. and C.G.) will extract data independently using a standardized form. Data will be reported in a Microsoft Excel (version 14.1.0, Redmond, WA: Microsoft, 2011) spreadsheet. The data collection form will be piloted with three articles. Modifications will be applied if variables are deemed superfluous or additional data should be collected, and if clarifications are needed. In case of discordance, a third party (E.B) will be consulted.

### Data items

The following data will be extracted from the studies: (1) Study characteristics: author, country in which the study was conducted, year of conduction and publication, study design, sample size; (2) Sample characteristics: maternal age, gestational age at amniocentesis, indication for amniocentesis, therapeutic intervention, fetal sex, gestational age at delivery, birthweight, outcome of pregnancy (PTL, PPROM, chorioamnionitis, microbial invasion of the amniotic cavity, admission to neonatal intensive care unit); (3) Exposure: inflammatory markers measured, definition of intra-amniotic inflammation; (4) Outcome: method for assessing neurodevelopment, children’s age at the time of neurodevelopmental evaluation, brain imaging; (5) Results of individual studies: measures of association between intra-amniotic inflammation and neurodevelopmental outcome (relative risks and adjusted relative risks), confounders adjusted for, association between inflammation and abnormal brain imaging.

### Outcomes and prioritization

Our primary outcome is child neurodevelopment, assessed either with the use of a validated questionnaire or by revision of medical records. Neurodevelopmental outcomes may include measurements of behavioral development, intelligence quotient, diagnosis of cerebral palsy, learning disabilities, etc. As studies related to this specific topic are uncommon, accepting different methods of neurodevelopment assessment (e.g., Bayley III scale, Stanford Binet Intelligence Scale, Rapid Neurodevelopmental Assessment, Ages and Stages Questionnaire, and Brief Infant-Toddler Social and Emotional Assessment) will enable us to include a larger number of studies in our systematic review. When neurological development is measured using a scale, if possible, it will be dichotomized as favorable or unfavorable neurodevelopmental outcome. Secondary outcomes will be abnormal brain imaging (e.g., white matter damage, periventricular leukomalacia), evaluated either with cerebral MRI or transfontanelle ultrasound. Abnormality will be defined according to the publication.

### Risk of bias in individual studies

The risk of bias of individual studies will be assessed with the Cochrane Collaboration’s tool for assessing risk of bias in randomized trials or an adapted version of the Risk Of Bias in Non-randomized Studies (ROBINS-1), a tool to assess risk of bias in non-randomized studies [30]. A study will be considered at high risk of bias when one domain qualifies as such. All studies, regardless of their methodological quality and risk of bias, will be eligible to the meta-analysis. However, sensitivity analyses will be performed after exclusion of studies with a high risk of bias.

### Risk of publication bias

We will assess the risk of publication bias by visual inspection of funnel plots.

### Data synthesis

Characteristics of included studies will be detailed, and relative risks and/or adjusted relative risks between intra-amniotic inflammation and neurodevelopmental outcomes will be reported with their 95% confidence intervals. The various dimensions of neurological development will be pooled separately. We will stratify analyses according to scales used to measure the neurodevelopmental outcomes. Relative risks will be pooled using the Mantel-Haenszel method and DerSimonian and Laird random effects and presented in a forest plot. When adjusted measures of association are reported, adjusted relative risks will be pooled with an inverse variance method and DerSimonian and Laird random effects. If available, means and standard deviations of neurodevelopment scores will be used to compute mean differences between groups and pooled using inverse variance with random effect models. Additionally, if concentrations of intra-amniotic inflammatory markers are available for groups of patients with favorable and unfavorable outcomes, mean differences of inflammatory markers will be pooled similarly.

Higgins’ *I*^2^ statistics will be used as an indicator of heterogeneity. Sensitivity analyses will be performed excluding studies with high risk of bias. If a sufficient number of studies are eligible to these analyses (at least three studies by subgroups), we will conduct subgroup analyses for term vs. preterm (delivery < 37 weeks’ gestation) neonates, gestational age at amniocentesis, interventions (e.g., anti-inflammatory or antibiotics) vs. no-intervention, fetal sex, and indication for the amniocentesis, including the diagnosis of PTL and/or PPROM at the time of amniocentesis.

Cochrane Review Manager (version 5.1, The Cochrane Collaboration, 2011) will be used to perform the analyses. If a meta-analysis cannot be performed due to substantial heterogeneity in exposures or outcome definitions, a qualitative summary of the results of eligible studies will be reported.

## Discussion

While amniotic inflammation has been strongly associated with severe obstetrical and neonatal outcomes, very few studies assessed its impact on long-term health of children, and few preventive strategies have been developed in this regard. To our knowledge, no previous systematic review has been conducted on this topic. Despite a rigorous methodology, we expect to find a small number of studies. We expect that most studies included women with PTL or PPROM rather than asymptomatic women in early pregnancy (for instance, at the time of a genetic amniocentesis); subgroup analyses will have to be interpreted cautiously. We may observe important heterogeneity in the chosen markers of intra-amniotic inflammation, definition of intra-amniotic inflammation, and methods used for assessing the neurodevelopmental outcome, possibly precluding performance of meta-analyses. However, we expect that similar inflammatory markers or similar outcomes will allow pooling, which will improve the power of the analyses.

Recent evidences suggest that an individualized approach, including specific antibiotics and inflammation suppressors, could have a positive effect on pregnancy and neonatal outcomes [27, 31]. Our systematic review will provide an exhaustive assessment of the current literature and a better understanding of the current evidence. Therefore, a better understanding of the association between amniotic inflammation and neurodevelopment, and modifiers of this association will help inform future research. The review could be the first step towards the development of new hypotheses on the pathological pathways leading to adverse neurodevelopmental outcomes after intra-amniotic inflammation.

## Additional file

Additional file 1: PRISMA-P 2015 Checklist. (PDF 159 kb)

## Abbreviations

PPROM: Preterm premature rupture of membranes
PTL: Preterm labor
ROBINS-1: Risk Of Bias in Non-randomized Studies
